# The Overwhelming Challenge of Losing One’s Sense of Wholeness: A Grounded Theory Study on Women’s Experiential Process of Birth Trauma

**DOI:** 10.1097/jnr.0000000000000741

**Published:** 2026-05-19

**Authors:** Li-Li CHEN, Wan-Lin PAN, Chin-Hsing TSAI, Yu-Chan LI

**Affiliations:** 1 Department of Nurse-Midwifery and Women Health, National Taipei University of Nursing and Health Sciences, Taipei, Taiwan, ROC; 2 School of Nursing, National Taipei University of Nursing and Health Sciences, Taipei, Taiwan, ROC; 3 Department of Nursing, Chang Gung University of Science and Technology, Taoyuan, Taiwan, ROC; 4 Department of Thanatology and Health Counseling, National Taipei University of Nursing and Health Sciences, Taipei, Taiwan, ROC

**Keywords:** childbirth, post-traumatic stress, perinatal care, experiential process, grounded theory

## Abstract

**Background:**

The physical and emotional experiences women undergo during childbirth influence not only the formation of their maternal role but also their willingness to have additional children. Birth trauma can lead to a depressed mental state in the postpartum period, which further complicates breastfeeding and the development of the mother–infant bond.

**Purpose:**

In this study, the experiential process of women with the experience of birth trauma during labor and delivery is explored.

**Methods:**

Strauss and Corbin’s grounded theory and purposive sampling were employed. Fifteen postpartum women were recruited, and face-to-face interviews were conducted from August 1 to November 30, 2024. A semistructured interview guide was used, and data analysis was performed using the constant comparative method from grounded theory.

**Results:**

Grounded theory was used to explore the experiential process of women who underwent birth trauma. The core category “overwhelming challenge of losing one’s sense of wholeness” that emerged from the narratives of the participants encompassed five subcategories, including unimaginable pain, loss of dignity, ignored emotions, unease from invasion of bodily privacy, and endless waiting. The outcome category was “I do not want to experience another childbirth.”

**Conclusions/Implications for Practice:**

The results of the exploration in this study of the process underlying the trauma experience of women during labor and delivery highlight the urgent need to improve the quality of care provided during labor. Obstetric clinicians and health care administrators may apply the theoretical model developed in this study to design appropriate continuing education programs and intervention strategies that, for example, implement shared decision-making, provide individualized care, and offer diverse methods for pain relief. It is hoped these findings will contribute to enhancing clinical practices, improving the quality of labor and delivery, and fostering more positive childbirth experiences for women.

## Introduction

Symptoms of postpartum traumatic stress have a profound impact on women during the postpartum period that hinders the establishment of the maternal role and often leaves lasting emotional scars. Recently, these symptoms have emerged as a significant international public health issue, with estimates suggesting that up to 45% of new mothers experience some form of trauma (Shorey & Wong, 2022). The findings of studies conducted in various countries indicate postpartum post-traumatic stress disorder (PP-PTSD) affects as many as 5.9% of all postpartum women (Suarez & Yakupova, 2023). Despite their prevalence, peripartum events and conditions such as PP-PTSD are generally insufficiently discussed or addressed.

Humenick (2006) describes childbirth as a “flow experience,” a transformative process that challenges women to grow and evolve in life-expanding ways. This process empowers women to feel control and mastery over their destiny, providing excitement and deep enjoyment while creating a milestone in their memories and a high point in their life (Csikszentmihalyi, 1991). However, research has also shown that childbirth can pose a threat to the life of the mother as well as of the fetus, making it a potential trigger for traumatic events that may lead to the development of PP-PTSD (Watson et al., 2021).

Studies have shown that 9%–44% of women experience trauma during childbirth and, in more-severe cases, they may develop the symptoms of PP-PTSD. The results of prior research also indicate women with high-risk pregnancies to be more likely to develop these symptoms, with an incidence rate of about 15%–18% (Dumpa & Kamity, 2019; Horsch et al., 2024).

In the United Kingdom, some 30,000 new cases of birth trauma are reported each year, with more than 30% of women experiencing birth trauma-related symptoms (Royal College of Obstetricians and Gynaecologists, 2023). As a result, the Royal College of Obstetricians and Gynaecologists and the National Institute for Health and Care Excellence have trained professional teams and established associations to support women affected by birth trauma (Royal College of Obstetricians and Gynaecologists, 2023). Influenced by the British Birth Trauma Association, Australian scholars founded the Australasian Birth Trauma Association in 2016 to address the issue in Australia (Australasian Birth Trauma Association, 2016).

In recent years, alongside the United Kingdom and Australia, there has been growing global research on birth trauma. Watson et al. (2021) conducted a scoping review to examine the factors contributing to birth trauma and propose strategies for addressing it. Their findings indicate women who experience traumatic childbirth events not only face a higher incidence of birth-related injuries but also suffer long-term psychological effects. Birth trauma has been shown to trigger persistent psychosocial symptoms, including anxiety, tokophobia (fear of childbirth), interpersonal relationship difficulties, and PTSD.

Furthermore, birth trauma may significantly influence the future reproductive decisions of these women and potentially deter them from having additional children (Watson et al., 2021).

Systematic reviews and meta-analyses have shown PP-PTSD to be linked primarily to events that occur during childbirth (Lai et al., 2023). Based on previous research, several risk factors for PP-PTSD have been identified, including prior trauma, fear of labor, low social support, and unexpected cesarean sections. Among these, negative subjective childbirth experiences represent the most significant risk factor (Ayers et al., 2016). A woman’s birth experience is influenced not only by communication and support from health care professionals but also by the personal significance of childbirth to her as well as her family background and cultural beliefs (Watson et al., 2021). Women’s subjective perceptions of childbirth also play a crucial role in their involvement in decision-making during labor (Hernández-Martínez et al., 2020; Martínez-Vazquez et al., 2021).

Birth trauma induces significant psychological changes in women (Nelson, 2024) that affect their mental health and parenting behaviors as well as the subsequent development and social relationships of their children (Molloy et al., 2021). Postpartum depression, the most common mental health condition associated with birth trauma (Ertan et al., 2021), has become a public health concern that requires proactive and careful attention. Therefore, it is essential to focus on maternal mental health and provide appropriate support to mitigate the negative effects of labor. Most previous studies conducted in Taiwan have focused on psychological distress during pregnancy (Wang et al., 2022) and postpartum intervention strategies (Sari et al., 2023), with relatively little attention paid to the psychological experiences of these women during the intrapartum period. In this study, qualitative interviews were used to explore the experiences and perceptions of women of their postpartum birth trauma during labor and delivery.

## Methods

### Study Design

The goal of grounded theory is to develop a model that accurately represents social phenomena (Strauss & Corbin, 1990). Grounded theory emphasizes that the model should be grounded in data collected and analyzed from the field, with a particular focus on understanding human interactions, processes, and social trajectories. Therefore, in-depth interviews were employed in this study to explore the trajectory of birth trauma. This study was approved by the institutional review board of National Taiwan University (approval number: 202309EM017).

### Setting and Participants

Postpartum women who gave birth at community-based obstetric clinics in northern Taiwan from August 1 to November 30, 2024, were recruited as participants. These clinics do not employ midwives, and care was provided by obstetricians and nursing staff. Qualitative interviews were conducted using open-ended questions that focused on subjective experiences of childbirth rather than the influence of specific provider roles. Although the care model was medically oriented, participants were able to freely describe their experiences within this obstetrician-led, medically oriented care context. After confirming the eligibility of potential participants, the investigator explained the study objectives, motivation, and interview process to the participants. Informed consent was obtained, and the consent form was signed before conducting face-to-face interviews.

Purposive sampling was employed. The inclusion criteria for participants were (a) birth given in a medical institution, (b) vaginal delivery (including those who experienced an unexpected cesarean section), (c) able to read and write in Chinese and complete the questionnaire, (d) able to communicate in Mandarin or Taiwanese and willing to participate in the interview, and (e) agree to participate in this study within 3–10 days postpartum after being informed of the objectives and procedures.

The exclusion criteria were (a) having a cognitive impairment or congenital mental illness or being unable to communicate verbally, (b) stillbirth or abnormal fetal genetic conditions, and (c) a score of >9 on the Edinburgh Postnatal Depression Scale. This threshold was used because scores ≤9 indicate a healthy postpartum psychological status.

Purposive sampling was used at the outset of participant recruitment, followed by theoretical sampling once a preliminary theory was established. Theoretical sampling commenced after the fifth participant had been enrolled. In addition to meeting the inclusion criteria, it was necessary that participants perceived their labor experience as having brought negative feelings and had thoughts of not wanting to experience labor again. The target sample size was 15 participants. Recruitment continued until no new themes emerged from the analyzed data, indicative of data saturation, at which point participant enrollment was closed.

### Data Collection

The instrument used in this study included the semistructured interview questions in Table 1. During the interviews, a voice recorder was used to enhance data authenticity and completeness. Data collection was initiated through referrals from postpartum care staff at postpartum facilities and medical institutions. After each participant signed an informed consent, a one-on-one interview was conducted. The investigator used open-ended questions to allow participants to describe their experiences retrospectively, and an interview outline was used to explore additional topics that had not been addressed following a semistructured interview format.

**Table 1:** Interview Questions

No.	Question
1.	Can you describe your experiences and feelings during the labor and delivery process?
2.	Were there any moments during labor or delivery when you felt particularly uncomfortable?
3.	Have you ever heard the term “birth trauma”?
4.	Since giving birth, do you find yourself feeling down or upset when reflecting on your labor and delivery experience?

### Data Analysis

Data analysis was conducted using the constant comparative method of grounded theory (Strauss & Corbin, 1998). Grounded theory involves three main coding procedures: open, axial, and selective. This iterative process begins with a line-by-line analysis followed by discussions with the researchers to identify key concepts and categories, which are then named. These initial concepts and categories are organized into tables and supplemented with memos (Corbin & Strauss, 2015).

During the analysis, the data were organized using computer word processing software (Microsoft Word and Excel; Microsoft, Redmond, WA, USA). Through ongoing comparison and categorization, the meanings expressed by the participants were identified. All of the memos were reviewed, organized, and integrated into the analysis. The verbatim transcripts were re-examined to ensure the identification of core and subcategories, which were then linked with existing categories to explore potential interrelationships, ultimately connecting them systematically with other categories.

### Rigor and Trustworthiness

Lincoln and Guba (1985) proposed that qualitative research must meet certain criteria, including credibility, transferability, dependability, and confirmability, to assess its reliability and validity. Rigor and trustworthiness were ensured through the use of individual semistructured interviews, which involved asking participants a consistent set of questions using a standardized interview guide designed to maintain neutrality. The one researcher who conducted all of the interviews holds a PhD in obstetrics, has received specialized training in qualitative research methodologies, and possesses 10 years of clinical experience in obstetric care. This individual’s integration of academic credentials, methodological expertise, and extensive field experience strengthened the study’s credibility and dependability and further reduced the risk of contamination by personal opinions or biases during data collection. To ensure validity, all interview data were transcribed verbatim without the addition of reliability annotations, and the researcher conducted data coding by repeatedly reviewing the transcripts. To ensure confirmability (verifiability), the final version of the interview summaries and thematic analysis was evaluated by two other experts (both PhD-qualified obstetric qualitative researchers with publication experience in the field).

## Results

### Study Sample and Demographics

The participants were all between 26 and 40 years old. Thirteen held college degrees, and two held master’s degrees. Five reported holding religious beliefs. The participants’ EDPS scores ranged from 0 to 6, with a mean of 3. In addition, two did not receive pain-relieving anesthesia during childbirth.

### Findings From Qualitative Interviews

The phrase “overwhelming challenge of losing one’s sense of wholeness” refers collectively to the experience of the participants during childbirth, where they were not treated as autonomous individuals but rather as vehicles for delivering a child. In this process, they endured immense hardship, feeling both challenged and, at times, powerless. This experience represented the core category of the trauma encountered during labor and delivery.

**Figure 1:**
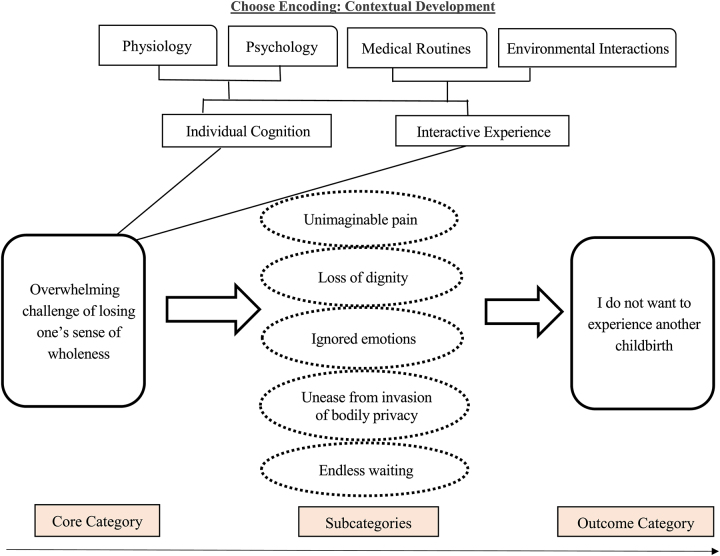
Model of the Childbirth Trauma Process

Among the subcategories, the experience of unimaginable pain during labor was prominent. Women were required to endure the pain of uterine contractions for the sake of their fetus. Also, many reported experiencing loss of dignity, ignored emotions, unease due to the invasion of bodily privacy, and endless waiting during the obstetric care process. The cumulative negative impact of these subcategories led to the outcome category of not wanting to experience childbirth again. From the analysis, it became clear that birth trauma was a negative experiential process with both a clear beginning and an end (at the conclusion of the childbirth process). However, the emotional and psychological impact of the experience remained deeply etched in memory of these individuals, making it unforgettable. The overall theoretical framework of this study is shown in Figure 1.

#### Unimaginable Pain

Childbirth involves the contraction and compression of the uterus, with the fetus moving downward and passing through the birth canal. This physiological process places tremendous pressure on the body, disrupting its original structure while ultimately leading to the completion of childbirth. Pain from childbirth is a major trauma-inducing factor. Its unpredictable intensity and the actual pain experience were often the most significant factors influencing the development of trauma in the participants.

*I kept feeling intense swelling and pain down there, along with cold sweats. The pain was so severe that I felt like I was about to faint*. (3)

*Throughout the entire experience, I couldn’t even cry. The pain was so overwhelming that I couldn’t manage to cry*. (4)

*I could clearly feel a part of my body being forcefully stretched open, and it was extremely painful, almost like scratching. I still remember that sensation, and even two or three days after giving birth, just thinking about it would make my scalp feel numb. I felt like I could never endure that again*. (6)

*Of course, if the pain had continued like that, I probably wouldn’t have been able to bear it. At one point, the pain felt so intense, it made me feel like I was dying. It also made me feel as though I wanted to die*. (7)

*There was a dull ache, and I started hitting the bed. I hit the bed because the pain was so intense, and I thought, “Why is this happening again?”* (13)

#### Loss of Dignity

Under the immense pressure of uterine contractions, women are deeply concerned with whether they are treated with respect. Respect, in this context, is defined by having access to information about the progress of labor, the ability to exercise informed consent, and the ability to maintain their physical appearance and sense of self, which affirms their identity as a person.

*My water was broken artificially, and I wasn’t informed beforehand. My husband was standing next to me, and then many medical staff, including a resident physician and two nurses, came in. They didn’t inform me in advance, and I felt very uncomfortable with this.* (1)

*The moment I was shaved was really shocking because the nurses were doing it carelessly, just shaving randomly, like they didn’t know what they were doing. It felt like they were just scraping away at something.* (2)

*The nurse was rude. When I had to move from the delivery table to the hospital bed, I asked, “Can you take the baby away first?” I just wanted the baby to be placed back in the crib and taken out of the room. But the nurse replied, “I can’t help you. Just keep holding the baby.”* (2)

*When I was giving birth, I felt like I looked ugly and completely unlike myself. That alone was deeply traumatic. I believe it is more traumatic than the pain itself. The most humiliating point in life was the day I gave birth.* (5)

*I just let the nurse do whatever she wanted to me. I felt powerless, and all I could do was let them treat me however they liked.* (6)

*Two nurses suddenly asked me to practice breathing. When I kept doing it wrong, they scolded me. I was scolded repeatedly.* (12)

*I didn’t know when he was going to come. When he did, he just said, “I need to examine you.” During contractions, they checked how dilated I was, so it was always an internal exam at the beginning to see how far I had dilated. He just pulled back my bedside curtain, and I thought, “Here we go again, another internal exam.”* (13)

#### Ignored Emotions

When undergoing immense physical pressure, women require psychological care. More than simply a process, childbirth is a significant event. While the health of the child is crucial, women are concerned to an even greater degree with having someone able to support, accompany, and reassure them, to let them know they are not alone.

*When pressure was applied to the lower part of my uterus, I remember a person standing on my right side, with their palm pushing down on my ribs repeatedly. I was awake, watching them do this to me, but I couldn’t say no. I just didn’t have the strength and couldn’t give birth at that moment. I felt like I was a mother at the mercy of others.* (1)

*The nurse performed an internal examination first, then the resident doctor did another internal examination. I kept going through repeated internal exams, and then the attending doctor came in for another one. The way they conducted these exams felt very forceful and violent.* (3)

*When I was having labor pains, I felt extremely uncomfortable. It felt as though I was being poked down there.* (7)

*During the intense pain leading up to childbirth, no one was able to help me. At that moment, the people around me couldn’t ease my pain, yet everyone thought it was just normal pain. No one seemed to care about me.* (8)

*Then they started pressing on my stomach. One nurse pressed hard on my stomach with her hands, and another may have been assisting the doctor. I was holding my breath desperately, and my face turned blue to the point that it made me nauseous.* (12)

*During the internal exam, my cervix hurt so much. The nursing staff said they were massaging the cervix to speed up labor, but I don’t know if it really helped. All I could do was cry. I kept thinking, “No, please don’t do this again,” but I held it in and didn’t say anything.* (12)

*It felt like my body wasn’t my own anymore. In the time leading up to childbirth, he came in and said, “I need to perform an internal exam.” It felt like my vagina was being examined without my control. He would leave, then come back again for more exams, and I couldn’t say no. It always felt like my body wasn’t mine.* (13)

*When he pressed on my stomach, it felt like I couldn’t breathe. He pressed on my stomach and uterus, pushing down on me, and I couldn’t catch my breath. He used his elbows to apply pressure, then pressed his entire body onto me. It felt like a wrestler was on top of me. When I was in pain, he told me to inhale, hold my breath for 10 seconds, and push with my stomach. While he was pressing on my stomach, I felt like I wanted to push him away. I was nearly out of breath as his whole body pressed down on me.* (14)

#### Unease from the Invasion of Bodily Privacy

Evaluation is crucial during the labor and delivery process. In hospital settings, internal exams are necessary to assess the progress of labor. Vaginal examinations are, therefore, an unavoidable part of the experience for women. In addition to enduring physical pain, women remain highly concerned about their privacy during labor and delivery. The exposure of intimate areas often causes a deep sense of unease that not only leads to feelings of humiliation but also contributes to psychological trauma.

*The lower half of my body was exposed, and the rest is as you can imagine. I didn’t want my husband to see the moment when my water broke, because there were two nurses holding my feet, and a female doctor was in front of me. It felt so direct, even though they said, “Let’s expose it.” My husband was standing next to me, and I really had no control over the situation. I was just so embarrassed.* (1)

*I felt like it was very undignified to have my legs spread open for the internal examination, and then to have them kept open for so long… It just made me realize that the hardest part of giving birth as a woman is actually having to spread your legs.* (2)

*My lower body had to be completely exposed, and a maternity pad was placed underneath. The pad had worn down so much that it was rubbing against my skin, and it became really uncomfortable.* (12)

#### Endless Waiting

Whether receiving pain-relieving anesthesia or not, the long and seemingly endless wait for birth not only depletes a woman’s physical energy but also causes significant psychological distress. While childbirth naturally requires time, the absence of adequate information or support during this waiting period transforms women’s expectations of childbirth into feelings of suffering and helplessness.

*It maybe took to 12 hours, from 9 am until that evening. When labor pain progressed slowly like that, it really felt like the whole day was dragging on… I felt like I wanted to end my life. In that moment, I had this overwhelming urge to escape and just wanted to give up on the idea of having the baby altogether.* (2)

*When time drags on, it’s really agonizing for pregnant women and mothers who are about to give birth. I remember thinking, “Why am I only dilated to two fingers wide?” Why did it take so long to reach just two fingers? It was very discouraging, and I felt disheartened when I learned that my dilation had only increased by half a finger’s width.* (4)

*There’s no progress. You just wait endlessly, and you don’t know when the end will come.* (15)

#### Outcome Category: “I Don’t Want to Experience Another Childbirth”

Pain of labor, loss of dignity, lack of privacy, feelings of loneliness, and the long waiting process all contribute to psychological trauma in women waiting to give birth, leading to subsequent refusal to undergo another childbirth experience.

*I will never give birth again after this kind of experience.* (1)

*The birth trauma made me not want to have a second child. The pain I felt at that moment left me exhausted, and I still remember how tired I felt at the time.* (4)

*I don’t want to go through this again. I’m really sure I don’t want to experience it again. After all, for me, it wasn’t a pleasant process, and I don’t want to repeat it.* (6)

Using methodology based on grounded theory, the experiential process of birth trauma in this study was analyzed, as shown in Figure 1.

## Discussion

Grounded theory was employed in this study to explore the process of birth trauma. The core category “overwhelming challenge of losing one’s sense of wholeness” ran through the entire process, with subcategories building upon one another. This accumulation of experiences encourages women to perceive childbirth as a difficult and overwhelming challenge, leading to birth trauma and, in the case of the participants, making them unwilling to undergo another childbirth.

It is essential to center the family and treat women as autonomous individuals in obstetric care to ensure they do not view themselves as mere vehicles for childbirth. This approach is especially important in the context of care provided during labor and delivery, which is when women should be able to experience and feel the changes in their bodies and in the fetus in their womb as they transform into mothers. Experiencing childbirth in this manner can provide women with a fresh start afterward.

Labor pain is the greatest threat perceived by women during childbirth (Chabbert et al., 2020). With advancements in medical care, the use of epidural anesthesia has become a common method for alleviating pain during labor (Halliday et al., 2022). In this study, the participants reported holding high expectations of pain relief through anesthesia during labor and delivery. When they were unable to use pain-relieving anesthesia as expected (e.g., due to rapid labor progression) or when the effectiveness of anesthesia was inadequate, they experienced the childbirth process as trauma. This unbearable pain was one of the primary reasons for the reluctance reported by the participants to undergo another pregnancy and childbirth.

In addition, the results identified that women may also experience birth trauma due to disrespect from medical staff, the arbitrary exposure of their private parts, and verbal abuse. The World Health Organization advocates for respectful care during pregnancy and childbirth that includes maintaining dignity, privacy, and confidentiality, ensuring freedom from harm and abuse, and promoting informed decision-making and continuous support during labor and delivery (World Health Organization, 2014). However, it is challenging for busy clinical staff to establish relationships with women in labor, much less provide emotional support, companionship, and continuous care. The large number of necessary medical interventions, including frequent internal exams, the administration of labor-inducing drugs, shaving, and episiotomy, increases workload and often forces medical staff to rush through their delivery room responsibilities. This can negatively affect the relationship between women experiencing labor and the health care team, making it difficult to provide high-quality, individualized care.

The findings of prior systematic research recommend that clinical medical teams and care providers be trained to incorporate changes in values ​​and attitudes, offer professional interpersonal communication, improve privacy in maternity wards, and establish quality improvement teams (Bohren et al., 2020). With appropriate training, the quality of clinical care may be improved while ensuring women receive dignified care (Landry et al., 2023).

Unexpected labor progression and prolonged waiting can deplete the physical and mental energy of women experiencing labor, thus closely associating the experience with traumatic childbirth (Chabbert et al., 2020). In this study, some of the participants experienced trauma due to the inadequate alleviation of key pain factors, while others receiving pain-relieving anesthesia lost patience with the prolonged waiting times. It is critical that women receive relevant information about their labor and/or anticipatory guidance from professional health care providers to ensure labor, rather than just a prolonged and aimless waiting period, is an experience filled with assurance and anticipation for the arrival of a newborn. These areas of professional knowledge and interpersonal interaction require the continuing education of obstetric care providers to ensure the best care model (Wang et al., 2022). Memories and trauma associated with childbirth can affect a patient’s overall health. Also, the physical and psychosocial changes related to pregnancy and childbirth influence postpartum maternal roles and family functioning. Therefore, providing adequate emotional and physical care during both labor and the postpartum period is essential to ensuring women have positive memories and emotions when reflecting on their childbirth experience (Ozcalik & Aksoy, 2024).

The factors that trigger birth trauma may be avoided through the provision of comprehensive care. Lawal et al. (2024) used qualitative research to emphasize the importance of understanding women’s expectations and facilitating two-way communication during childbirth (Lawal et al., 2024). By encouraging women’s voices and allowing them to express their needs during labor, these crucial needs may be addressed in a safe environment (Isobel, 2023). Therefore, by exploring the process through which birth trauma is formed, this study was designed to identify effective mitigation strategies to reduce the underlying causes of this type of trauma.

### Strengths and Limitations

The strength of this study lies in its use of grounded theory to guide the qualitative interview process. In analyzing and organizing participant narratives, several key themes were identified, after which they were integrated with existing empirical research to develop a trauma process model. This novel conceptual model may be applied and further refined by researchers in other areas. It is hoped that future research will lead to the development of a birth trauma scale based on this trauma process model for clinical application.

One key limitation of this study relates to potential influence bias due to the interviewer. While standardized procedures and interviewer training were implemented, the tone, phrasing, and nonverbal cues used during the interviews may have shaped participant responses and, in turn, affected data authenticity. Despite careful efforts to ensure consistency, the influence of this nature is difficult to fully eliminate in qualitative research.

Another limitation is the limited external validity of this study due to the specific cultural and clinical context of the sample. This research was conducted in community-based obstetric clinics in northern Taiwan, which may not reflect the full range of postpartum experiences in other regions. Differences in cultural perceptions of childbirth and local obstetric care practices may limit the broader applicability of these findings. In future studies, more diverse samples from across Taiwan should be included to strengthen the generalizability of the findings and conclusions.

### Conclusions

This study was designed to examine the trauma related to maternity care and its impact on the emotions of women experiencing labor. As labor progresses, it is essential for medical staff to center care on their patient and their families by providing help with shared decision-making as well as showing respect to their patients and to their physical and emotional needs. This approach can help improve the memories and satisfaction of women regarding their childbirth experience. In line with the findings, health care providers and midwives should, regardless of whether pain relief is used during childbirth or not, make every effort to understand the needs of their patients and support them throughout labor. During this process, the utmost dignity should be afforded to women experiencing labor, and holistic care should be integrated into obstetric practice. Looking ahead, a domestic birth trauma scale may be developed to effectively screen for and prevent trauma during the postpartum period.

## References

[R1] Australasian Birth Trauma Association. 2016. *Birth trauma* https://birthtrauma.org.au/our-story/

[R2] Ayers S. Bond R. Bertullies S. Wijma K. 2016. The aetiology of post-traumatic stress following childbirth: A meta-analysis and theoretical framework. Psychological Medicine, 46(6), 1121–1134. 10.1017/S0033291715002706 26878223

[R3] Bohren M. A. Tunçalp Ö. Miller S. 2020. Transforming intrapartum care: Respectful maternity care. Best Practice & Research Clinical Obstetrics & Gynaecology, 67, 113–126. 10.1016/j.bpobgyn.2020.02.005 32245630

[R4] Chabbert M. Panagiotou D. Wendland J. 2020. Predictive factors of women’s subjective perception of childbirth experience: A systematic review of the literature. Journal of Reproductive and Infant Psychology, 39(1), 43–66. 10.1080/02646838.2020.1748582 32475156

[R6] Corbin J. Strauss A. 2015. Basics of qualitative research. Sage Publications, Inc.

[R7] Csikszentmihalyi M. 1991. [Book review] Flow, the psychology of optimal experience. American Journal of Psychotherapy, 45, 142–143. 10.1176/appi.psychotherapy.1991.45.1.142

[R8] Dumpa V. Kamity R. 2019. Birth trauma. StatPearls.

[R9] Ertan D. Hingray C. Burlacu E. Sterlé A. El-Hage W. 2021. Post-traumatic stress disorder following childbirth. BMC Psychiatry, 21(1), Article No. 155. 10.1186/s12888-021-03158-6 33726703 PMC7962315

[R10] Halliday L. Nelson S. M. earns R. J. 2022. Epidural analgesia in labor: A narrative review. International Journal of Gynaecolology and Obstetrics, 159(2), 356–364. 10.1002/ijgo.14175 35277971

[R11] Hernández-Martínez A. Rodríguez-Almagro J. Molina-Alarcón M. Infante-Torres N. Rubio-Álvarez A. Martínez-Galiano J. M. 2020. Perinatal factors related to post-traumatic stress disorder symptoms 1–5 years following birth. Women and Birth, 33(2), e129–e135. 10.1016/j.wombi.2019.03.008 30954482

[R12] Horsch A. Garthus-Niegel S. Ayers S. Chandra P. Hartmann K. Vaisbuch E. Lalor J. 2024. Childbirth-related posttraumatic stress disorder: definition, risk factors, pathophysiology, diagnosis, prevention, and treatment. American Journal of Obstetrics and Gynecology, 230(3S), S1116–S1127. 10.1016/j.ajog.2023.09.089 38233316

[R13] Humenick S. S. 2006. The life-changing significance of normal birth. The Journal of Perinatal Education, 15(4), 1–3. 10.1624/105812406X151330

[R14] Isobel S. 2023. Trauma and the perinatal period: A review of the theory and practice of trauma-sensitive interactions for nurses and midwives. Nursing Open, 10(12), 7585–7595. 10.1002/nop2.2017 37775971 PMC10643851

[R15] Lai X. Chen J. Li H. Zhou L. Huang Q. Liao Y. Krewski D. Wen S. W. Zhang L. Xie R. H. 2023. The incidence of post-traumatic stress disorder following traumatic childbirth: A systematic review and meta-analysis. International Journal of Gynaecology and Obstetrics, 162(1), 211–221. 10.1002/ijgo.14643 36571476

[R16] Landry I. René C. Demontigny F. 2023. Family centered nursing practices towards women and their families in the birthing context: A qualitative systematic review. Nursing Open, 10(9), 5937–5949. 10.1002/nop2.1880 37306178 PMC10416028

[R17] Lawal T. Dodge L. E. Toffey D. Zera C. Wu M. Larson E. 2024. Facilitating positive birth experience when preferences are not met: A qualitative analysis. Birth, 51(2), 275–283. 10.1111/birt.12783 37876307

[R18] Lincoln Y. S. Guba E. G. 1985. Naturalistic inquiry. Sage Publications.

[R19] Martínez-Vazquez S. Rodríguez-Almagro J. Hernández-Martínez A. Delgado-Rodríguez M. Martínez-Galiano J. M. 2021. Long-term high risk of postpartum post-traumatic stress disorder (PTSD) and associated factors. Journal of Clinical Medicine, 10(3), Article 488. 10.3390/jcm10030488 33573115 PMC7866544

[R20] Molloy E. Biggerstaff D. L. Sidebotham P. A. 2021. phenomenological exploration of parenting after birth trauma: Mothers perceptions of the first year. Women and Birth, 34(3), 278–287. 10.1016/j.wombi.2020.03.004 32303461

[R21] Nelson H. O. 2024. Experiencing birth trauma: Individualism and isolation in postpartum. Social Science & Medicine, 345, Article 116663. 10.1016/j.socscimed.2024.116663 38364723

[R22] Ozcalik H. B. Aksoy Y. E. 2024. The relationship between maternal functioning and birth memory and trauma. Midwifery, 132, Article 103974. 10.1016/j.midw.2024.103974 38503117

[R23] Royal College of Obstetricians and Gynaecologists. 2023. *NICE guidelines*. https://www.birthtraumaassociation.org.uk/for-health-professional/nice-guidelines

[R24] Sari Y. P. Hsu Y.-Y. Nguyen T. T. B. 2023. The effects of a mindfulness-based intervention on mental health outcomes in pregnant women: A systematic review and meta-analysis. The Journal of Nursing Research, 31(6), Article e306. 10.1097/jnr.0000000000000586 38036493 PMC11812663

[R25] Shorey S. Wong P. Z. E. 2022. Traumatic childbirth experiences of new parents: A meta-synthesis. Trauma Violence Abuse, 23(3), 748–763. 10.1177/1524838020977161 33256544

[R26] Strauss A. Corbin J. 1990. Basics of qualitative research grounded theory procedures and techniques. Sage Publications.

[R27] Strauss A. Corbin J. 1998. Basics of qualitative research: Techniques and procedures for developing grounded theory. Sage Publications.

[R28] Suarez A. Yakupova V. 2023. Past traumatic life events, postpartum PTSD, and the role of labor support. International Journal of Environmental Research Public Health, 20(11), Article 6048. 10.3390/ijerph20116048 37297652 PMC10252538

[R29] Wang T.-H. Tzeng Y.-L. Teng Y.-K. Pai L.-W. Yeh T.-P. 2022. Evaluation of psychological training for nurses and midwives to optimise care for women with perinatal depression: A systematic review and meta-analysis. Midwifery, 104, Article 103160. 10.1016/j.midw.2021.103160 34753017

[R30] Wang S.-W. Chen J.-L. Chen Y.-H. Wang R.-H. 2022. Factors related to psychological distress in multiparous women in the first trimester: A cross-sectional study. The Journal of Nursing Research, 30(3), Article e210. 10.1097/jnr.0000000000000485 35446283

[R31] Watson K. White C. Hall H. Hewitt A. 2021. Women’s experiences of birth trauma: A scoping review. Women and Birth, 34(5), 417–424. 10.1016/j.wombi.2020.09.016 33020046

[R32] World Health Organization. 2014. *The prevention and elimination of disrespect and abuse during facility-based childbirth*: *WHO statement*. https://iris.who.int/bitstream/handle/10665/134588/WHO_RHR_14.23_eng.pdf

